# Closed-loop automated critical care as proof-of-concept study for resuscitation in a swine model of ischemia–reperfusion injury

**DOI:** 10.1186/s40635-022-00459-2

**Published:** 2022-07-08

**Authors:** Nathan T. P. Patel, Eduardo J. Goenaga-Diaz, Magan R. Lane, M. Austin Johnson, Lucas P. Neff, Timothy K. Williams

**Affiliations:** 1 Department of Surgery, Wake Forest Baptist Medical Center, Hanes Building, B005, One Medical Center Boulevard, Winston-Salem, NC 27157 USA; 2 Division of Cardiac Anesthesiology, Department of Anesthesiology and Critical Care Medicine, Children’s Hospital of Philadelphia, Philadelphia, PA, USA; 3 Department of Cardiothoracic Surgery, Wake Forest Baptist Medical Center, Winston-Salem, NC USA; 4 Division of Emergency Medicine, University of Utah, Salt Lake City, UT USA; 5 Department of Pediatric Surgery, Wake Forest Baptist Medical Center, Winston-Salem, NC USA; 6 Department of Vascular/Endovascular Surgery, Wake Forest Baptist Medical Center, Winston-Salem, NC USA

**Keywords:** Closed-loop, Critical care, Automated, Ischemia reperfusion, Swine

## Abstract

**Background:**

Volume expansion and vasopressors for the treatment of shock is an intensive process that requires frequent assessments and adjustments. Strict blood pressure goals in multiple physiologic states of shock (traumatic brain injury, sepsis, and hemorrhagic) have been associated with improved outcomes. The availability of continuous physiologic data is amenable to closed-loop automated critical care to improve goal-directed resuscitation.

**Methods:**

Five adult swine were anesthetized and subjected to a controlled 30% estimated total blood volume hemorrhage followed by 30 min of complete supra-celiac aortic occlusion and then autotransfusion back to euvolemia with removal of aortic balloon. The animals underwent closed-loop critical care for 255 min after removal of the endovascular aortic balloon. The closed-loop critical care algorithm used proximal aortic pressure and central venous pressure as physiologic input data. The algorithm had the option to provide programmatic control of pumps for titration of vasopressors and weight-based crystalloid boluses (5 ml/kg) to maintain a mean arterial pressure between 60 and 70 mmHg.

**Results:**

During the 255 min of critical care the animals experienced hypotension (< 60 mmHg) 15.3% (interquartile range: 8.6–16.9%), hypertension (> 70 mmHg) 7.7% (interquartile range: 6.7–9.4%), and normotension (60–70 mmHg) 76.9% (interquartile range: 76.5–81.2%) of the time. Excluding the first 60 min of the critical care phase the animals experienced hypotension 1.0% (interquartile range: 0.5–6.7%) of the time. Median intervention rate was 8.47 interventions per hour (interquartile range: 7.8–9.2 interventions per hour). The proportion of interventions was 61.5% (interquartile range: 61.1–66.7%) weight-based crystalloid boluses and 38.5% (interquartile range: 33.3–38.9%) titration of vasopressors.

**Conclusion:**

This autonomous critical care platform uses critical care adjuncts in an ischemia–reperfusion injury model, utilizing goal-directed closed-loop critical care algorithm and device actuation. This description highlights the potential for this approach to deliver nuanced critical care in the ICU environment, thereby optimizing resuscitative efforts and expanding capabilities through cognitive offloading. Future efforts will focus on optimizing this platform through comparative studies of inputs, therapies, and comparison to manual critical care.

**Supplementary Information:**

The online version contains supplementary material available at 10.1186/s40635-022-00459-2.

## Introduction

The cornerstone of shock management has relied upon support of hemodynamics to maintain tissue perfusion and resulting oxygen delivery. This has primarily relied upon vasopressors and volume optimization, outside of the needed intervention for source control in the patient with septic shock, arrest of hemorrhage in the bleeding patient, or coronary artery bypass/percutaneous coronary intervention in the patient with ischemic cardiogenic shock. The vital signs of a patient in shock are labile and require intensive care unit (ICU) admission for invasive hemodynamic monitoring. Additionally, close attention by healthcare personnel is necessary to provide frequent assessments and adjustments in response to a patient's changing condition. This involves manual titration of fluid and vasopressor infusions for the treatment of a patient with severe shock, which can be a time consuming process [1]. Also, the excessive cognitive load associated with these tasks, amplified across an ICU with numerous critically ill patients, can lead to errors, burnout, job dissatisfaction, and increased patient mortality [2–4].

Hemodynamic support in shock, centers around the goal of strict blood pressure control, which has been suggested to confer benefit in many shock etiologies [5–8]. While establishing target hemodynamic endpoints is straightforward, either based on standard practice or individualized goals, the real challenge in the context of a critically ill patient on numerous infusions and life support devices is to achieve and maintain these endpoints over time. Prior studies in ICU and operating room patients on vasopressors report 6.0–11.2% of time spent with a mean arterial pressure (MAP) < 60 mmHg [9, 10]. While in one study 60% of the time patients in an ICU on vasopressors had a MAP > 70 mmHg and in another, 40% of the time was spent with a MAP > 80 mmHg [9, 10]. This bias towards the hypertensive state suggests an intentional effort on the part of providers. This in part reflects an intentional permissive stance, where hypertension is deemed preferable over hypotension both from clinical outcomes perspective and from the realization that tight blood pressure targeting is inherently more labor intensive and more difficult to achieve. However, SEPSIS-PAM suggests that a bias towards achieving or allowing hypertension through the use of exogenous vasopressor administration does not improve overall (exception of patients with chronic hypertension and association with renal failure) and a meta-analysis did not identify any benefits to supratherapeutic use of vasopressors with increased risk of new-onset supraventricular tachycardia [11, 12]. In addition, there are small case series reporting on the rare but limb threatening complication of symmetric peripheral gangrene due to excessive vasopressor use [13].

It is certainly true that failure to achieve or maintain target hemodynamic goals is unavoidable in certain situations due to a host of factors. However, optimization of hemodynamics, specifically achieving target hemodynamic goals, can undoubtedly be achieved through improvements in clinical care. How to do so is a matter of debate, but it is increasingly recognized that automation utilizing closed-loop fluid and drug delivery is a viable and rational approach to solving this clinical dilemma [14, 15]. By combining ubiquitous continuous hemodynamic monitoring platforms, embedded microsystems to process these data using established rule sets or intelligent algorithms, and output devices that can administer or titrate fluids and drugs, many aspects of critical care management can be either partially or fully automated.

There are several benefits to an automated critical care platform to administer and titrate fluids and drugs. First, the time required to complete the cycle of recognition of clinical change, intervention, and clinical effect would be dramatically shorter. This allows for a strategy where smaller changes are made more frequently, leading to tighter control of hemodynamics. Second, the programming of a closed-loop control system effectively leads to standardization of care protocols, which allows best practices and safeguards to be implemented regardless of the ability or immediate availability of the bedside provider. Lastly, a closed-loop control system would likely decrease task load for bedside providers and improve provider bandwidth. In the setting of staff shortages or in austere environments this may allow expanded patient care capabilities.

There is recent interest using translational models for testing of closed-loop resuscitation paradigms for the titration of vasopressors during anesthesia and resuscitation of hemorrhagic shock [16–18]. Closed-loop automation of fluid and drug delivery has been examined in humans for treatment of burn resuscitation and blood pressure control under general anesthesia [19, 20]. Additionally, there have been feasibility studies exploring fully automated life support in healthy swine [21]. However, the development of an automated critical care platform for the management of shock states for use in the ICU environment has not been extensively explored. Our group is developing such a platform, specifically for use in vasodilatory shock [22]. We hypothesize that in a swine model of ischemia–reperfusion injury, an automated critical care platform can be developed that provides fully autonomous crystalloid and vasopressor titration based on a simple heuristic model of critical care. This would provide a testing platform for precise measurements and reproducible treatment for the study of various critical care strategies.

## Methods

### Overview

The Institutional Animal Care and Use Committee at Wake Forest Baptist Medical Center approved this study. All animal care and use were in strict compliance with the Guide for the Care and Use of Laboratory Animals in a facility accredited by AAALAC.

### Animal preparation

Healthy adult, castrate male and non-pregnant female, Yorkshire-cross swine (Oak Hill Genetics, Ewing, IL, USA) were fasted for 12 h before experimentation, then premedicated with 5–7 mg/kg intramuscular tiletamine/zolazepam (Telazol, Zoetic Inc. Olot, Spain). After isoflurane induction and endotracheal intubation, animals were maintained with ~ 2% isoflurane and mechanically ventilated to maintain end-tidal CO_2_ at 35–45 mmHg. All animals received a 1-L bolus of balanced electrolyte solution, followed by maintenance of intravenous fluids of 10 mL/kg/h (PLASMA-LYTE A, Baxter Healthcare Corporation, Deerfield, IL, USA). To offset the vasodilatory effects of isoflurane, an intravenous infusion of norepinephrine (0.02 mcg/kg/min) was instituted upon venous access and titrated before experimentation to achieve a target MAP > 60 mmHg. An underbody warmer was used to maintain core body temperature between 37 and 39 ºC. A laparotomy was undertaken for placement of a cystostomy tube and a splenectomy was performed to minimize hemodynamic variation from autotransfusion. The maintenance fluid rate was decreased to 5 mL/kg/h after abdominal wall closure.

### Instrumentation

A surgical cut-down approach was used to place a 7 Fr arterial sheath in the right common femoral artery and a 9 Fr arterial sheath in the left common femoral artery. A dual-lumen 9 Fr venous resuscitation line was placed in the left femoral vein for blood transfusion and resuscitation fluids. Left external jugular vein was surgically exposed and cannulated with a 9 Fr sheath, respectively, to allow for maintenance fluid, vasoactive medication administration, and central venous pressure measurements. A 7 Fr arterial sheath was placed in the left axillary artery after surgical exposure for proximal blood pressure measurements. The right brachial artery was exposed and cannulated with a 5 Fr sheath to facilitate frequent lab draws. A 7 Fr custom compliant aortic balloon catheter was introduced through the left femoral 9 Fr arterial sheath and positioned at the level of the diaphragm. The position of the aortic balloon catheter was confirmed in the distal descending thoracic aorta by manual palpation. Finally, the abdomen was closed with cable ties and plastic sheet to minimize insensible fluid losses. Intravenous heparin (50 units/kg bolus and 10 unit/kg/h continuous rate) was administered to achieve an activated clotting time of ~ 100 s based on a pre-calculated rate heuristically developed from prior studies to prevent complications of intra-aortic balloon placement (Additional file 1: Fig. S1).

### Data collection

Physiologic measurements of proximal blood pressure, distal blood pressure, and central venous pressure, were collected in real time with a multi-channel data acquisition system at a rate of 1000 Hz (Powerlab, AD Instruments, Colorado Springs, CO, USA).

### Experimental flow

After instrumentation, the animals underwent an automated computer-controlled 30% estimated total body blood volume controlled hemorrhage over 30 min and the shed blood was collected in gently agitated citrated bags (Fenwell Blood Collection, Fresenius Kabi, Bad Homburg, Germany) and stored in a warm water bath at 39 ºC. Estimated total blood volume in milliliters was calculated as 60 ml/kg. After hemorrhage, the prepositioned aortic occlusion balloon was autonomously inflated to complete occlusion as measured by the complete absence of distal arterial pulse pressure for 30 min. At T45 (during occlusion phase), the automated drug delivery system infused 200 mg of calcium gluconate over 20 min to counteract the anticipated transfusion of citrate from the autologous blood transfusion. At T55, the animals were returned to 95% of total blood volume over 18 min. Starting at T60 the aortic balloon was programmatically weaned over 15 min during transfusion to prevent complete hemodynamic collapse from reperfusion injury and then the aortic balloon was removed. The critical care phase lasted for a predetermined 255 min (5.5 h from start of experiment).

### Automated critical care system

**Fig. 1:**
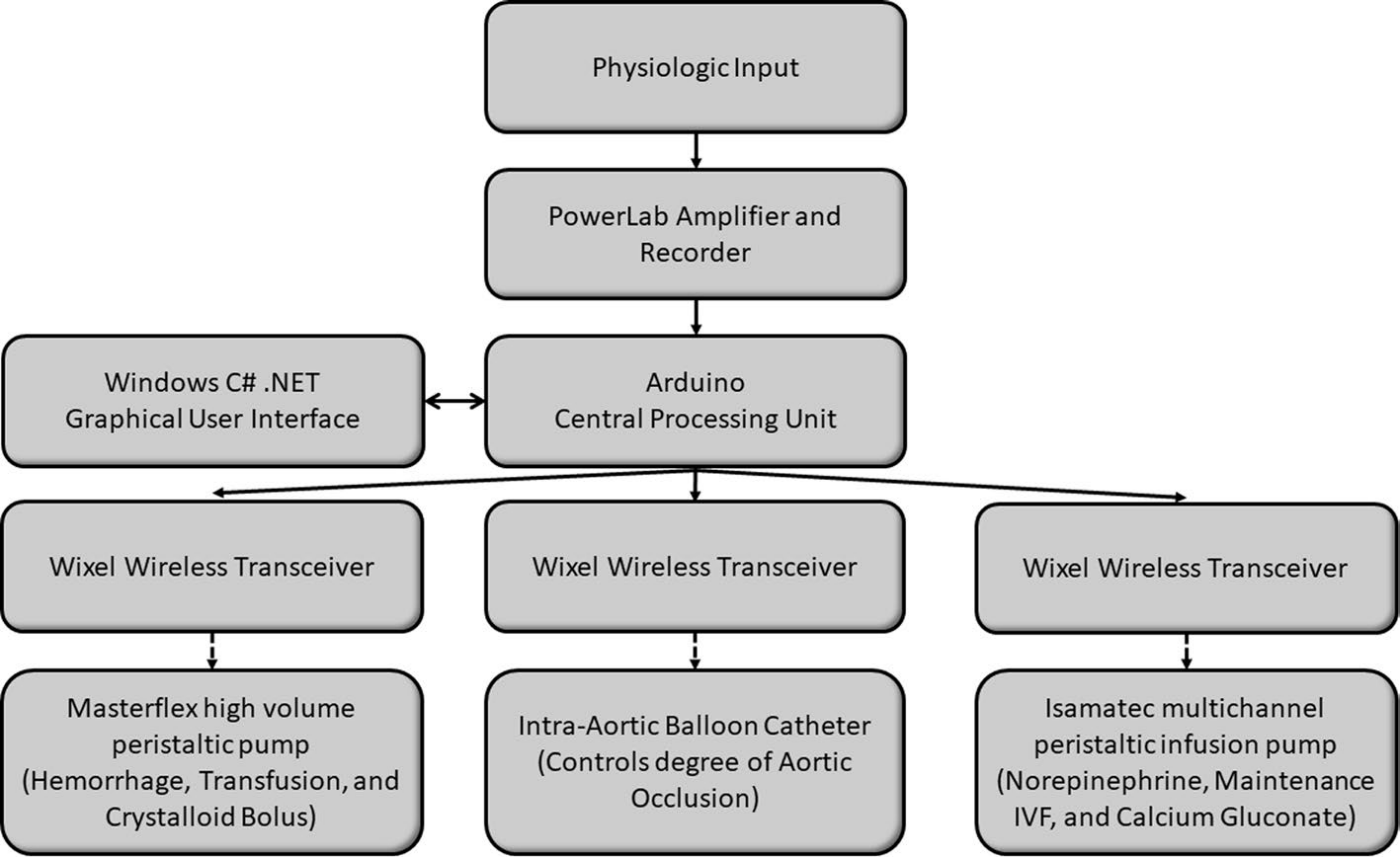
Diagram demonstrating the hardware setup and communication pathway. Solid arrow represents physical hardware connection and dotted arrow represents wireless communication

**Fig. 2:**
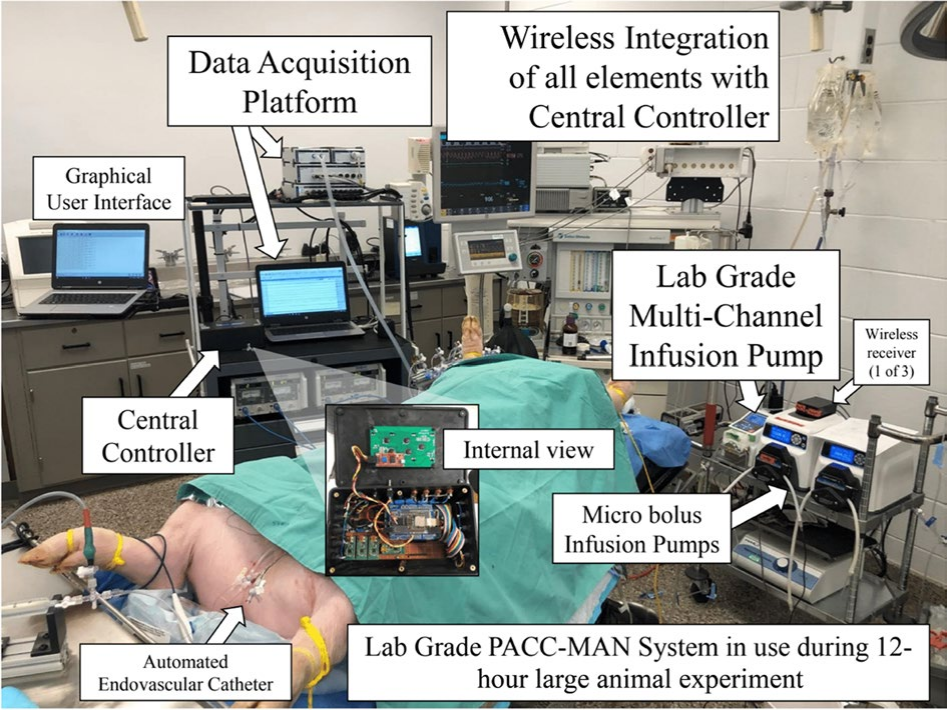
A picture of the setup in use for a critical care experiment. Data Acquisition platform (Powerlab, AD Instruments, Colorado Springs, CO, USA), Central Controlled (Arduino, Arduino LLC, Boston, MA, USA), Wixel Wireless Transceiver, Pololu Corporation, Las Vegas Nevada (Wireless receiver), Lab Grade multi-channel infusion pump (Isamatec, Cole-Palmer an Antylia Scientific Company, Vernon Hills, IL, USA), and Micro bolus Infusion Pump (Masterflex an Antylia Scientific Company, Antylia Scientific, Vernon Hills, IL, USA)

A custom-designed central processing unit was built using an Arduino embedded microsystem (Arduino LLC, Boston, MA, USA) for monitoring continuous analog data output from the physiologic recording system (Power Lab, AD Instruments, Colorado Springs, CO, USA). A custom graphical user interface was developed using C#.NET (Windows Corporation, Redmond, Washington, USA) to display real-time blood pressure measurements and to execute logic for control of peripheral devices (step-by-step pump controls). Additionally, a novel builder functionality was developed to carefully choreograph events throughout the experiment, enabling replication of experimentation with millisecond precision. Using three wireless transceivers (Pololu Corporation, Las Vegas, NV, USA), bidirectional communication was established to control a multi-channel peristaltic pump (Isamatec, Antylia Scientific Company, Vernon Hills, IL, USA), high-volume peristaltic pump (Masterflex, Antylia Scientific, Vernon Hills, IL, USA), and custom-built balloon syringe pump [22]. This created a closed-loop physiology monitoring, injury creation, interpretation, and intervention system. See Fig. 1 for schematic representation of the hardware setup and Fig. 2 for an example of the system in use during an experiment.

### Standardized critical care algorithm

Algorithms for drug and fluid delivery and titration were designed based on observations from multiple prior swine studies and heuristics of critical care for the management of shock. This iteration is an improvement on the original algorithm used for development of combined resuscitation with endovascular perfusion augmentation study [23]. The main inputs include arterial blood pressure, central venous pressure, current norepinephrine dose, and number of crystalloid boluses. Crystalloid boluses were given as weight-based (5 mL/kg, i.e., for a 70-kg pig corresponds to 350 mL over 2 min which corresponds to an infusion rate of approximately 10.5 L/h). This combined automated system and algorithm has been termed Precision Automated Critical Care Management (PACC-MAN). See Additional file 1: Fig. S2 for a complete diagram of the critical care algorithm.

Continuous proximal mean aortic blood pressure is measured and the average for the last 1 min is continuously calculated. If the animal has an average mean arterial blood pressure of 60–70 mmHg (inclusive), then unless the animal is on a large dose of norepinephrine (> 0.5 mcg/kg/min) and a low central venous pressure (< 6 mmHg) the animal does not receive an intervention. If the animal has an average mean arterial blood pressure over 1 min > 70 mmHg, then this is an opportunity to decrease vasopressors.

Vasopressor titration is performed using an algorithm that factors in the absolute difference between current MAP and target MAP of 65 mmHg, square root of current norepinephrine rate, scaling factor (determined based on branch in algorithm, integers like 1 or 2), and scaling constant (0.0072). The function is still relatively linear in the normal ranges of mild hypotension and mild hypertension.

The majority of the complexity is borne out in the hypotension algorithm with two main branches. The first branch, denoted “Severe Hypotension” (MAP < 50 mmHg), triggers a weight-based fluid bolus (5 mL/kg) and a simultaneous vasopressor dose increase. The second branch, denoted “Moderate Hypotension” (MAP 50–59 mmHg, inclusive) is more nuanced. This involves an algorithm for the estimation of “fluid responsiveness”. “Fluid responsiveness” is generally defined as a cardiac output augmentation of 10–15% in response to crystalloid therapy and a change in blood pressure is commonly used to assess a hypotensive patient's cardiac output response to a fluid challenge [24, 25]. While the efficacy of blood pressure change’s correlation to cardiac output augmentation is debated, it is a reliable input for building a closed-loop critical care algorithm. Therefore, a preload-dependent state was defined as a change in blood pressure in response to a weight-based test bolus (5 mL/kg) over 2 min. A “responsive” state was defined as an increase in MAP of at least 5 mmHg. Additional interventions are based on this binary classifier. In general, the “fluid responsive” state involves a more fluid-avid resuscitation, whereas the “fluid non-responsive” state involves a more vasopressor-avid resuscitation. There are specific corner cases for the “fluid non-responsive” states to (1) avoid cyclic increases in norepinephrine (“CVP-based Fluid Bolus”) and (2) attempt a fluid bolus for animals on maximum pressors, hypotensive, and not volume responsive (“Pressor Resistance Algorithm”). This algorithm was based on heuristics from numerous animals that have undergone this specific injury.

Every four interventions or after an hour since the last fluid responsiveness test bolus, the algorithm resets to “unknown” and an additional test bolus is administered in response to the next episode of hypotension.

### Data analysis

Data analysis was performed with Python Version 3.7 (Python Software Foundation, Wilmington, Delaware, USA), Excel 2016 (Microsoft Corporation, Redmond, Washington, USA), and R statistical software (R Foundation for Statistical Computing, Vienna, Austria). Due to anticipated small sample size values are presented as medians with interquartile range. Based on our review of the literature and severity of this injury model the goal of the algorithm was to avoid hypotension of 90% while having < 20% of the time spent at hypertension.

Hypotension was defined as MAP of < 60 mmHg, hypertension as MAP of > 70 mmHg, and normotension as MAP of ≥ 60 mmHg and ≤ 70 mmHg. 1000-Hz data were binned into 1-min blocks of time.

A priori exclusion criterion was a white blood cell count > 25 10^9^/L or expiration before the end of study as defined by a MAP < 20 mmHg for 5 min during any time point (including instrumentation). Also if norepinephrine at a dose rate of > 0.1 mcg/kg/min was required for more than 10 min cumulative duration during setup or if norepinephrine at a dose rate of > 0.06 mcg/kg/min was required to maintain normotension immediately prior to the beginning of the experiment (time zero).

## Results

**Fig. 3:**
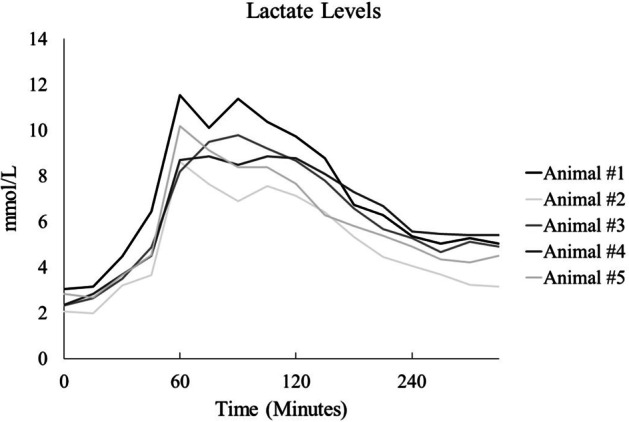
Lactate levels throughout the experiment. At 30 min it was the end of controlled hemorrhage. At 60 min it was the end of ischemia. At 75 min was the start of critical care

There were 8 animals available for analysis and underwent standardized critical care after ischemic perfusion injury. One animal was excluded due to protocol violation (error with pump rates), one animal had injury protocol violations and then died early in experiment, and one animal died later during the critical care phase. A complete list of baseline characteristics can be seen in Table 1. The median peak lactate after injury was 9.77 mmol/L (interquartile range: 8.86–10.17 mmol/L) and the median lactate at end of experiment was 4.91 mmol/L (interquartile range: 4.51–5.04 mmol/L). See Fig. 3 for lactate levels throughout the experimental protocol.

**Table 1:** Baseline characteristics and injury metrics

Results	Median with interquartile range
Number of animals	8
Number of excluded animals	3
Females of included animals	3
Weight (kg)	70.9 (65.4–78.8)
Initial pH	7.426 (7.4–7.43)
Initial Hgb (g/dL)	9.9 (9.9–10.5)
Initial WBC (g/dL)	16.99 (16.29–20.07)
Initial Cr (mg/dL)	1.8 (1.5–1.8)
Initial BUN (mg/dL)	9 (8–9)
Initial K (mmol/L)	3.7 (3.7–3.7)
Initial glucose (mg/dL)	95 (89–102)
Baseline proximal mean arterial pressure (mmHg)	63.2 (62.34–67.72)
Injury phase: proportion of time with distal hypotension (< 62.5 mmHg)	98.3% (83–100%)
Injury phase: proximal mean arterial pressure at end of hemorrhage phase (mmHg)	30.15 (28.73–41.64)

**Fig. 4:**
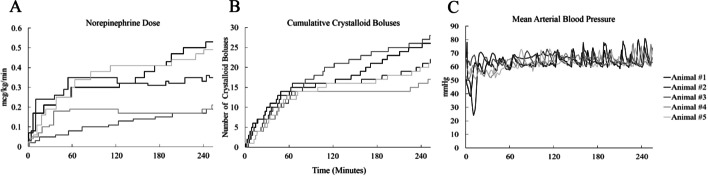
The norepinephrine dose rate (**A**), number of crystalloid boluses (**B**), and mean arterial pressure (**C**) during the critical care phase

During the 255 min of the critical care phase, the animals experienced hypotension (< 60 mmHg) 15.29% (interquartile range: 8.63–16.47%) of the time, hypertension (> 70 mmHg) 7.84% (interquartile range: 6.67–9.02%) of the time, and normotension (60–70 mmHg) 76.86% (interquartile range: 76.86–81.18%) of the time. During the first 60 min of the critical care phase (immediately following the injury phase) the proportion of time spent at normotension was 56.67% (interquartile range: 35.00–56.67%), while time at hypotension was 46.33% (interquartile range: 35.00–65.00%) with negligible time at hypertension 0% (interquartile range: 0–0%). When examining the critical care phase beyond the first 60 min (the latter part including 155 min of critical care), the animals experienced normotension 83.08% (interquartile range: 83.08–87.18%) of the time, hypotension 1.03% (interquartile range: 0.51–7.69%) of the time, and hypertension 10.00% (interquartile range: 8.72–11.79%) of the time. Figure 4 is a visual representation of the mean arterial blood pressure throughout the experiment. Table 2 provides a breakdown for each animal and their proportion of time at specific blood pressure ranges.

**Table 2:** Proportion of time at specific blood pressure ranges (ranges are inclusive unless otherwise denoted with less than or greater than sign) using 1-min bin to calculate mean arterial blood pressure during the critical care phase (255 min)

	Animal #1 (%)	Animal #2 (%)	Animal #3 (%)	Animal #4 (%)	Animal #5 (%)
< 60 mmHg	16.9	8.6	5.1	15.3	26.3
60–70 mmHg	76.5	77.3	85.5	80.8	66.7
70–80 mmHg	6.7	13.3	9.4	3.9	7.1
> 80 mmHg	0	0.8	0	0	0

The median intervention rate was 8.47 interventions per hour (interquartile range: 7.76–9.18 interventions per hour). The interventions were proportioned as 61.54% (interquartile range: 61.11–66.67%) weight-based crystalloid boluses and 38.46% (interquartile range: 33.33–38.89%) titration of vasopressors (increase or decrease). Figure 4 provides a plot of the norepinephrine dose and number of weight-based crystalloid boluses throughout the course of the experiment.

## Discussion

In a swine model of ischemia–reperfusion injury causing vasodilatory shock, this autonomous critical care algorithm using closed-loop control of vasopressor titration and crystalloid boluses was able to provide hemodynamic support using mean arterial pressure and central venous pressure as inputs to avoid hypotension 84.72% (normotension 76.86% and hypertension 7.84%) of the time. In addition, this algorithm using a rule-based heuristic model of critical care was able to provide frequent interventions during the first hour after injury for the “active resuscitation” and then transition to long-term critical care with goal-directed blood pressure management, avoiding hypotension 93.08% of the time.

This translational model is severe and one animal did die early in the experiment, but that animal also had protocol violations during the injury model potentially making the injury more severe and another animal died later in the experiment and unclear the cause as it was sudden. The initial severe hypotension after removal of endovascular balloon may be unavoidable with traditional critical care adjuncts (pressors and crystalloid). The main application of this system at this time would be to have a form of “standardized critical care” from which to test hypotheses in translational models of differing novel adjunctive critical care interventions. Although this model of critical care could provide the groundwork for developing clinically testable algorithms and hardware solutions to assist with off-loading cognitive load for the clinician and nursing staff. The potential for closed-loop critical care in the operating room and in the intensive care unit as a clinical support tool for monitoring and intervening is certainly far away at this point, but the field is progressing quickly with the ever increasing availability of real-time digital healthcare information.

The main limitations of this model are sample size (5 swine) and duration of critical care (255 min). This was not a survival study and therefore long-term implications of this injury model and interventions are unknown. Also this algorithm did not have a direct comparison to a control group. This algorithm also may suffer from overfitting to this injury model and needs additional testing in a wider variety of shock models to verify its generalizability. Additionally, the potential instability of closed-loop inputs (blood pressures) in potential real-world scenarios was not tested. Finally, the granularity of “fluid responsiveness” is overly simplistic in this iteration (although blood pressure response is still widely used in intensive care settings). Our group is currently iterating refinements of our methodology to assess fluid responsiveness based around the concept of augmentation of cardiac output: a machine learning model, near-infrared spectroscopy, continuous cardiac thermistor, and mixed central venous spectroscopy to potentially more accurately measure the gradations of fluid responsiveness as opposed to a simple binary classifier based on blood pressure [24, 26, 27].

Autonomous control systems for use in medical applications has gained significant interest in the broad literature, the potential for closed-loop control is quickly becoming technically feasible, but not without reasons for concern. Among them are control system failure, automation bias, skill degradation, lack of operational transparency, and increased risk arising from system complexity [28]. Additionally, there is legitimate concern from a medico-legal perspective regarding liability in the context of adverse outcomes attributable to failures involving automated interventions. There are additional regulatory hurdles to developing autonomous platforms, particularly those that involve actuation of devices or control drug and fluid delivery. Furthermore, developing clinically relevant algorithms that can be generalized to the entire patient population and across disease states represents an obstacle, particularly given the protracted pace of clinical research. Ultimately, many hurdles still need to be crossed to deliver these types of autonomous therapies from bench to bedside. But, our ongoing federally funded research aims to develop a commercially viable prototype which is slated to occur over the next 3 years.

The next steps in refinement of the PACC-MAN platform will center around additional inputs to the decision tree, to include metabolic measures such as lactate and urine output. Additionally, we plan to augment the complement of interventions to include additional vasopressor agents (i.e., vasopressin) and to refine the implementation of existing interventions, ultimately to improve the fidelity of the model and to optimize physiologic endpoints of resuscitation. Finally, future work will directly compare this platform to human control alone, as well as assessing its efficacy in longer term experiments and survival models.

## Conclusion

This autonomous critical care platform, utilizing volume expansion and vasopressor titration in a model of ischemia–reperfusion injury, using a goal-directed closed-loop algorithm based on heuristics of critical care provides a platform for prospective testing of critical care adjuncts in the treatment of circulatory failure. This descriptive technique can provide the framework for a patient-specific and “just enough” approach to critical care. Future directions include testing against manual critical care, variations of diagnostic/treatment paradigms, and injury models.

## Supplementary Information

**Additional file 1: Figure S1**. Instrumentation anatomy. **Figure S2. **Flow diagram of the algorithmic approach to critical care.

## Data Availability

The datasets used and/or analyzed during the current study are available from the corresponding author on reasonable request.

## Abbreviations

ICU: Intensive care unit
MAP: Mean arterial blood pressure
PACC-MAN: Precision automated critical care management
CVP: Central venous pressure
