# Supplementary material for: Closed-loop automated critical care as proof-of-concept study for resuscitation in a swine model of ischemia–reperfusion injury
Source: Intensive Care Med Exp. 2022 Jul 8;10:30. doi: 10.1186/s40635-022-00459-2 (PMC9263023; doi:10.1186/s40635-022-00459-2)

**Supplemental Figure 1.** Instrumentation anatomy

**
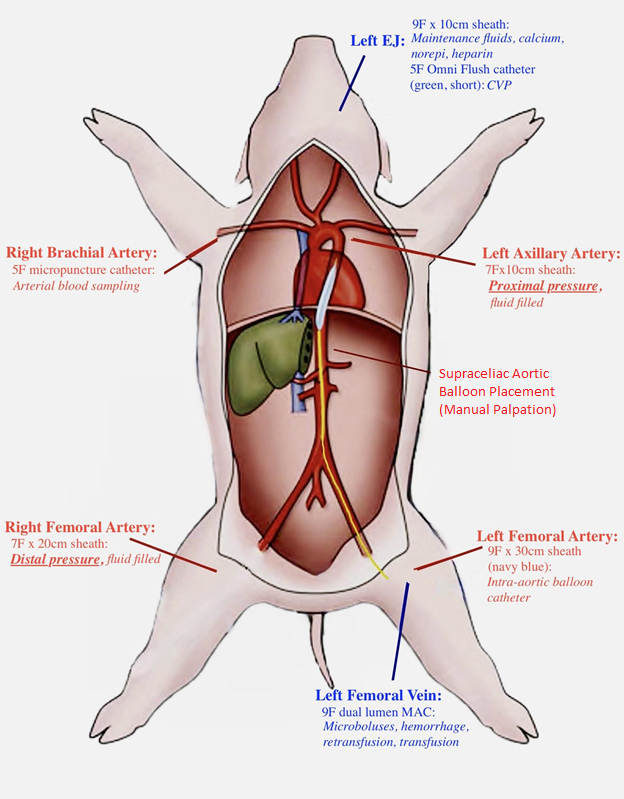
**

**Supplemental Figure 2.** Flow diagram of the algorithmic approach to critical care.


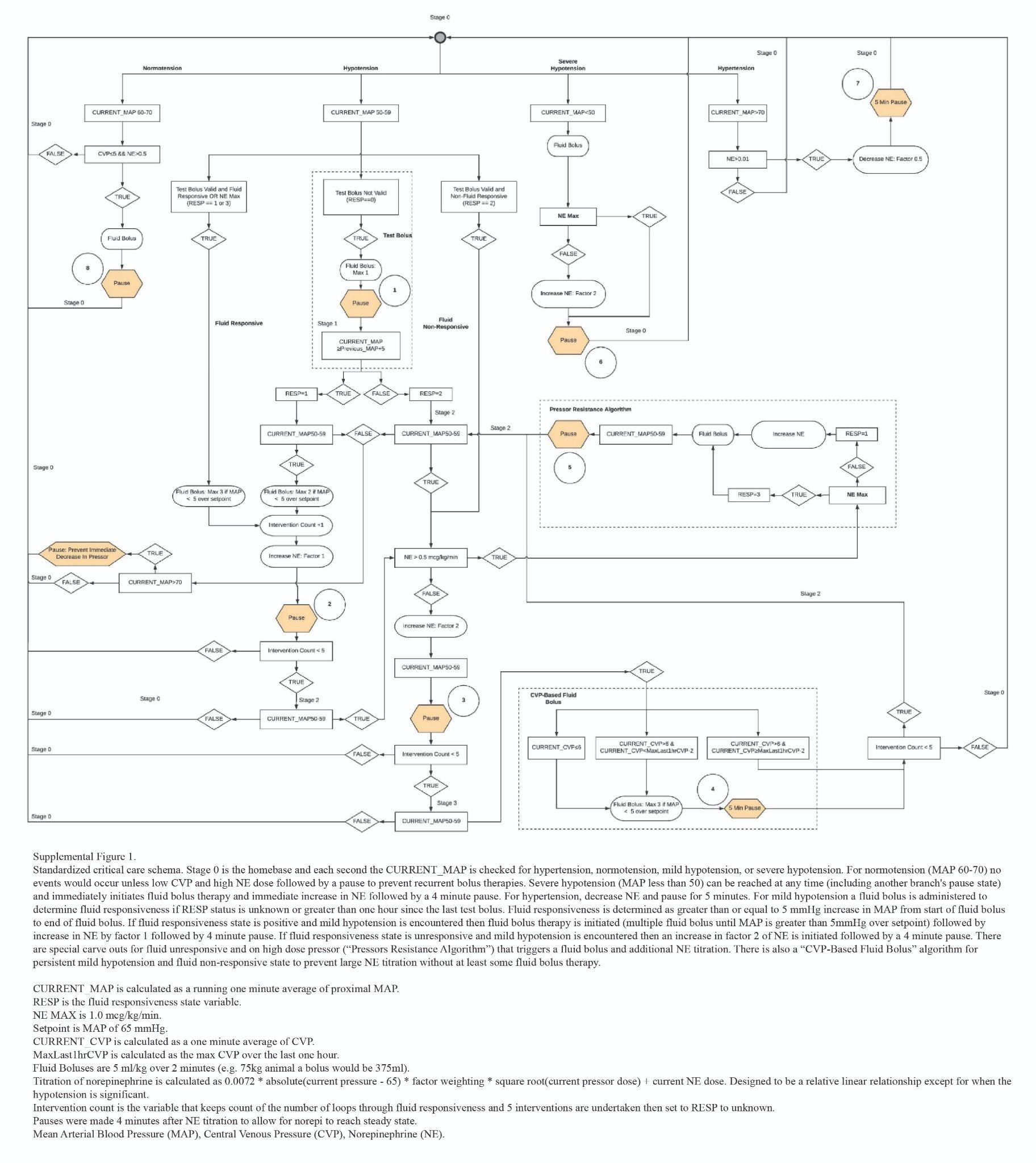

Supplement: Supplementary file 1 — Additional file 1: Figure S1. Instrumentation anatomy. Figure S2. Flow diagram of the algorithmic approach to critical care. [file 40635_2022_459_MOESM1_ESM.docx]
